# Isolated radial scar diagnosis by core-needle biopsy: Is surgical excision necessary?

**DOI:** 10.1186/s40064-016-1993-z

**Published:** 2016-03-31

**Authors:** Elizabeth Min Hui Kim, Andrea Hankins, Jamie Cassity, Dennis McDonald, Barbara White, Ron Rowberry, Sharon Dutton, Claire Snyder

**Affiliations:** Sutter Medical Group, Sacramento, CA USA; Johns Hopkins University Bloomberg School of Public Health, Baltimore, MD USA; Sutter Institute for Medical Research, Sacramento, CA USA; Diagnostic Pathology Medical Group, Sacramento, CA USA; Johns Hopkins University School of Medicine, Baltimore, MD USA

**Keywords:** Breast cancer, Radial scar, Radial sclerosis, Surgical management, Benign breast disease

## Abstract

**Purpose:**

Radial scar and radial sclerosis (RS) are considered benign breast lesions with proliferative features. There is sparse literature on frequency of cancer upgrade in these patients without atypical features found on image-guided needle biopsy. This study retrospectively reviews cases of isolated RS diagnosed on needle biopsy and evaluates the cancer upgrade after subsequent surgical excision.

**Methods:**

We conducted a retrospective cross-sectional study of cases with an isolated RS diagnosis based on needle biopsy and subsequent surgical pathology among all patients between January 1, 2009 and December 31, 2013. Patients with concomitant atypia, lobular carcinoma in situ on core biopsy, complete excision of very small RS with needle biopsy, and radiology-pathology discordance were excluded. An upgrade from the needle biopsy of RS was defined as surgical excision pathology that revealed ductal carcinoma in situ (DCIS), invasive ductal carcinoma (IDC), and/or invasive lobular carcinoma (ILC).

**Results:**

10,921 image-guided needle biopsy pathology reports were collected and 88 patients (0.81 %) were identified as having isolated RS. Of these 88 patients, 63 (72 %) underwent excision. The upgrade rate to cancer on subsequent surgical excision was 1.59 % (1/63) for DCIS; 0 % (0/63) for IDC; and 0 % (0/63) ILC. Twenty-five patients who did not undergo surgical excision had stable imaging studies with mean (±SD) 26 (±20) months follow up.

**Conclusions:**

Isolated radial scar on needle biopsy may not warrant routine surgical excision given relatively low cancer upgrade rates. Advancement in breast imaging, pathology and multidisciplinary approaches to care may effectively guide non-surgical management of RS.

## Background

Breast cancer is the most common cancer diagnosed and the second leading cause of cancer-related deaths in women in the United States (Howlader et al. 2011). Women with a history of benign breast disease (BBD) are at an increased risk for developing breast cancer. A well-established BBD classification system was developed by Dupont and Page, which consists of (1) non-proliferative disease, (2) proliferative disease without atypia, and (3) proliferative disease with atypia (Dupont and Page 1985). Dupont and Page, and others reviewed more than 10,000 benign breast biopsies and they found that women with BBD with atypia had a breast cancer relative risk 4.4 times higher than that of the general population (Dupont and Page 1985; Degnim et al. 2007; Morgan et al. 2012).

Quantifying the risk of breast cancer of proliferative disease without atypia has been an ongoing challenge (Degnim et al. 2007; Morgan et al. 2012; Krishnamurthy et al. 2012). The lack of consensus on management of these patients with benign and high risk breast disease can be demonstrated by a survey taken by the American Society of Breast Surgeons in 2011 (Krishnamurthy et al. 2012). Approximately 1020 practicing breast surgeons were asked about their preferred management of these breast lesions, including atypical lobular hyperplasia, atypical ductal hyperplasia, lobular carcinoma in situ (LCIS), flat epithelial atypia, radial scar and papilloma without atypia (Krishnamurthy et al. 2012). Surgical excision biopsy was recommended for most patients when needle core biopsy showed these high risk lesions were present. Surgical excisional biopsy was recommended in approximately 63 % of cases for papilloma without atypia, 75 % for atypical lobular hyperplasia, 70 % for LCIS, 72 % for flat epithelial atypia, and 76 % for radial scar (Krishnamurthy et al. 2012). These variations of practice pattern are due to lack of robust research outcome studies.

**Fig. 1:**
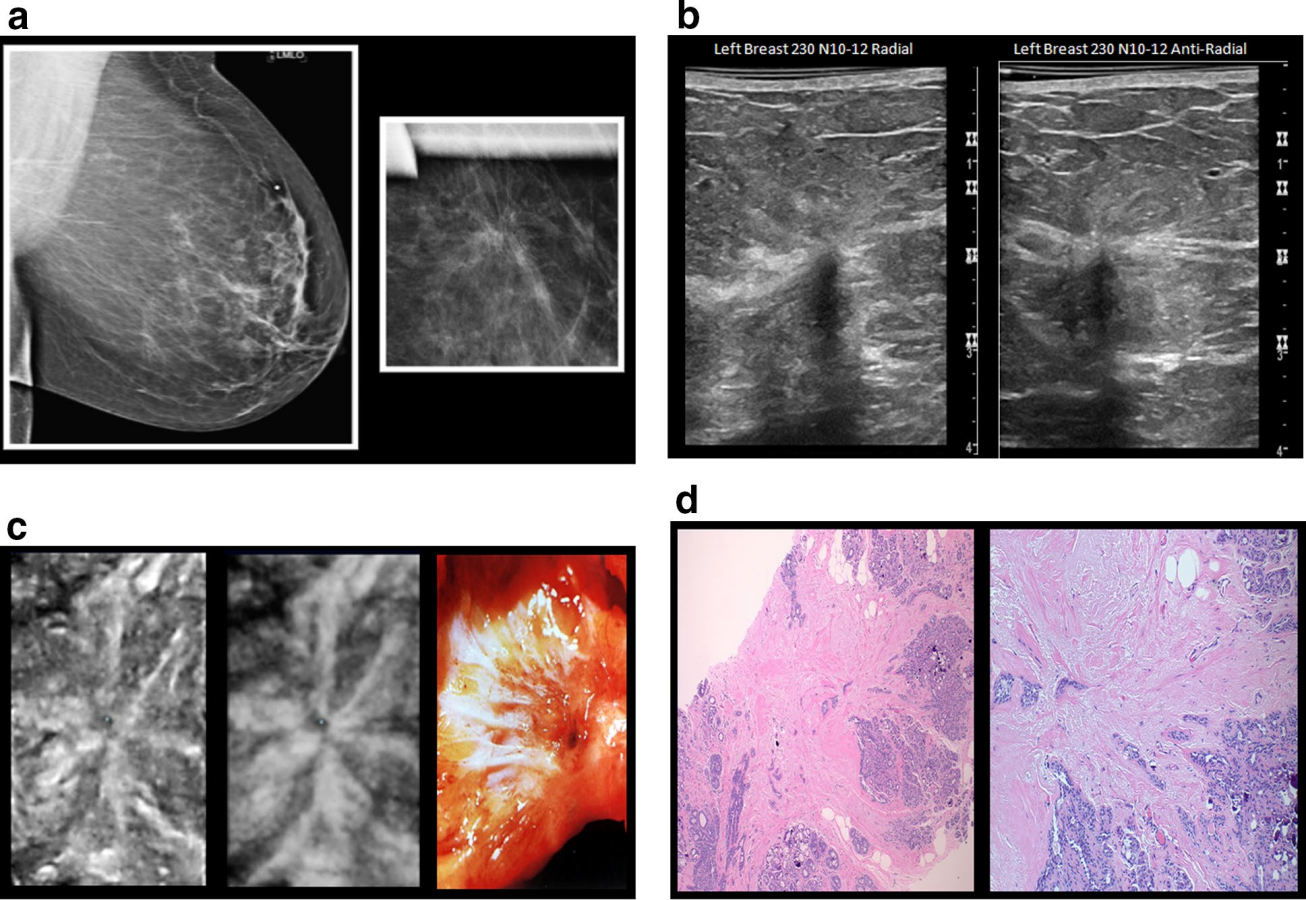
**a** Left breast screening mammogram and compressing view. Left breast mammogram shows non-palpable speculated lesion with radiating bands. They appear as an asymmetric density or area of architectural distortion without a central mass. Radiating spicules in a background of radiolucent fatty tissue create a “*black star*” appearance. **b** Ultrasound showing radial scar. Ultrasound shows ill-defined hypoechoic area with or without posterior shadowing and associated distortion (which is best demonstrated on coronal view). **c** Radial scar coronal plane. *From left to right*: *Left picture* shows ultrasound 1 mm thick slice of conventional imaging. *Middle picture* shows 2 mm thick slice showing hyperechoic fibroelastosis. *Left picture* shows gross appearance dominated by fibroelastosis. **d** Haematoxylin-eosin stain of radial scar core needle biopsy. Core needle biopsy pathology (original magnification ×100; haematoxylin-eosin stain) shows speculated radial scar with a central nidus of dense fibroelastotic tissue and radiating fibrous bands surrounded by “corona” of glandular proliferations and cysts

At our institution, there has been a growing interest in assessing patient outcome in the setting of a multidisciplinary breast team, and determining need for surgical excision of radial scar or radial sclerosis (RS) of the breast. As illustrated in Fig. 1, RS is a histological diagnosis characterized by an irregular spiculated pattern of epithelial proliferation radiation from a central fibroelastotic core with entrapped and distorted ducts (Morgan et al. 2012; López-Medina et al. 2006; Resetkova et al. 2011; Linda et al. 2010). Clinically, it is challenging to differentiate from carcinoma due to spiculated characteristics on imaging studies. In the modern era, percutaneous image guided core needle biopsy has essentially replaced upfront surgical biopsy for tissue diagnosis and to exclude malignancy. Controversy still exists regarding the need for surgical excision after a core needle biopsy diagnosis of isolated RS. Current literature on cancer upgrade rates is limited due to very small study sample size, study samples consisting of a mixture of both surgical biopsy and core needle biopsy cases, and study samples consisting of both isolated RS without atypia and RS with atypia (Krishnamurthy et al. 2012; López-Medina et al. 2006; Resetkova et al. 2011; Linda et al. 2010; Sohn et al. 2010; Doyle et al. 2007; Douglas-Jones et al. 2007; Berg et al. 2008; Becker et al. 2006; Cawson et al. 2003; Brenner et al. 2002). Therefore, these studies do not address the question of the surgical cancer upgrade rate of isolated RS after core needle biopsy. Many cancer institutions, similar to ours, resort to routine policy to recommend surgical excisional biopsy when RS is found on core needle biopsy to safely exclude malignancy. However, surgery is not without potential complications, and economic costs and benefits remain unclear.

## Purpose

The purpose of this study is to retrospectively review isolated RS diagnosis found on image-guided percutaneous core needle biopsies, and determine cancer upgrade after subsequent surgical excision.

## Methods

This study was reviewed and approved by the Sutter Health Central Area Institutional Review Committee (IRC). We also obtained administrative and institutional approval from the approved study site(s), including Roseville, Sacramento, Davis, Auburn, Amador, Solano, and Yuba City. Data was obtained through a query of the Sutter electronic medical record system (EPIC), and Diagnostic Pathology Medical Group (DPMG) electronic medical database (Claris) to establish the cases meeting the study inclusion criteria. Patient paper charts were retrieved to extract information not available in electronic medical records.

The study design was retrospective and observational. Our study utilized available aggregate database analysis from EPIC, DPMG and patient charts to obtain patient demographic and medical history. Adult females who underwent needle biopsy at Sutter Imaging Center from Jan 1, 2009 through Dec 31, 2013 were included. Core needle biopsy and surgical pathology results with diagnosis of isolated RS were analyzed. Exclusion criteria included male gender, previous ipsilateral cancer diagnosis 6 months prior to breast biopsy, patients with concomitant atypia or LCIS on core biopsy, complete excision of very small RS with core needle biopsy and radiology-pathology discordance. An upgrade from the core biopsy of isolated RS was defined as surgical excision pathology that revealed ductal carcinoma in situ (DCIS), invasive ductal carcinoma (IDC), and/or invasive lobular carcinoma (ILC).

To evaluate the impact of needle size and upgrade in cancer, the size (gauge) of the core needle and the type of image technology were noted. Breast lesions biopsied stereotactically were obtained with the use of the 14, 11 and 8 gauge vacuum assisted device (Hologic, Marlborough, MA). Stereotatic biopsies were performed while the patient lay prone on a dedicated stereotactic table (Hologic, Marlborough, MA). Ultrasound guided biopsy (Bard, Murray Hill, NJ) and Breast MRI guided biopsy (Hologic, Marlborough, MA) were used. After core samples were obtained, a small biopsy marker (Hologic, Marlborough, MA) was placed at the site of the biopsy.

A combined database containing demographic data, family history of breast cancer, alcohol/tobacco use, body mass index (BMI), pathology, and breast density was obtained by linking the EPIC/Clarity and DPMG/Claris databases by patient name and date of birth. Missing values were obtained by EPIC chart review. Cases of radial scar were filtered from the pathology report and validated by chart review.

Descriptive statistics were used to examine and explore the data. Chi square Test of Independence (categorical variables) and analysis of variance (continuous variables) were used to compare differences between the excision and non-excision groups. P values less than 0.05 were considered statistically significant.

## Results

We identified 10,921 image-guided core needle biopsy breast pathology reports from the years 2009 through 2013, of which 88 patients (0.81 %) were identified as having isolated RS diagnosis with radiology-pathology concordance. All 88 patients were recommended for surgical excision by the pathologist and radiologist. Of the 88 patients, 63 (72 %) underwent surgical excision, and 25 (28 %) did not undergo surgical excision after core biopsy.

As illustrated in Table 1, there were no statistically significant differences between the abstracted demographics of those who underwent excision versus those who did not. The mean age was 56 years in both groups. The majority of patients in both categories were self-reported to be White. The average BMI ranged from 27 (excision group) to 30 (non-excision group), both of which are classified as obese. Between 40 and 43 % of patients in each category reported as using alcohol. Among those who underwent excision, 59 % were “never smokers” versus 40 % among those who did not undergo excision. Almost all patients with reported mammographic breast density had >25 %. Among patients who underwent a surgical excision, 8 % reported a family history of breast and ovarian cancer, whereas none of the patients who did not undergo surgical excision reported family history.

**Table 1:** Subject characteristics and risk factors

Characteristic	Those who underwent surgical excision (n = 63)	Those who did not undergo surgical excision (n = 25)	P value
Age—n (%)			0.77
<39	3 (5)	1 (4)	
40–49	19 (30)	7 (28)	
50–59	17 (27)	8 (32)	
60–69	16 (25)	8 (32)	
>70	8 (13)	1 (4)	
Unknown	0 (0)	0 (0)	
Age—mean (SD)	56 (12)	56 (10)	0.84
Family history—n (%)			0.23
No	45 (71)	17 (68)	
Yes	5 (8)	0 (0)	
Unknown	13 (21)	8 (32)	
Race/ethnicity—n (%)			0.53
White	43 (68)	22 (88)	
Hispanic	0 (0)	0 (0)	
American Indian/Alaska Native	1 (2)	0 (0)	
Asian	1 (2)	0 (0)	
African American	2 (3)	0 (0)	
Filipino	1 (2)	0 (0)	
Unknown	15 (24)	3 (12)	
BMI—mean (SD)	27 (6)	30 (8)	0.19
Tobacco—n (%)			0.24
Current	4 (6)	3 (12)	
Never	37 (59)	10 (40)	
History	12 (19)	4 (16)	
Unknown	10 (16)	8 (32)	
Active alcohol use—n (%)			0.16
No	20 (31)	4 (16)	
Yes	27 (43)	10 (40)	
Unknown	16 (25)	11 (44)	
Density, mammographic—n (%)			0.29
<25 %	1 (2)	1 (4)	
>25–50 %	11 (17)	9 (36)	
>50–75 %	39 (62)	12 (48)	
>75 %	3 (5)	0 (0)	
Unknown	9 (14)	3 (12)	

Of those who did not undergo surgery, all lesions were non-palpable and were detected at the time of screening mammogram or breast MRI (Table 2). Four patients who underwent breast MRI were considered high risk patients with estimated life time risk of developing breast cancer (LTRBC) >20 %. As part of American Cancer Society Guidelines, these patients undergo breast MRI as adjunct to screening mammogram (Saslow et al. 2007). The size of abnormalities were all ≤20 mm (2 cm). The majority of the lesions were vacuum-assisted biopsies with size 9–11 g needle and, on average, 4–6 core tissue samples removed.

**Table 2:** Imaging and core biopsy characteristics of patients who did not undergo surgery

Initial detection imaging type	Indication	Type of abnormality	Size of abnormality (mm)
Mammogram	Screening (n = 21)	Cluster of calcifications (n = 9)	5–20
		Mass (n = 7)	7–17
		Architectural distortion (n = 2)	5–20
		Unknown (n = 3)	–
Breast MRI	High risk patient screening (n = 4)	Enhancing nodule (n = 1)	5
		Non-mass like enhancement (n = 1)	14
		Unknown (n = 2)	–

Needle size (n = 16)	Type of core biopsy needle	Number of core biopsy samples
14 gauge needle	2-Vac	4–6 cores
11 gauge needle	8-Vac	4–6 cores
9 gauge needle	6 (4-Vac and 2-nonVac)	4–6 cores
Unknown size (n = 9)	–	–

The cancer upgrade rate for both Invasive Ductal Carcinoma and Invasive Lobular Carcinoma was 0 %. One patient (1.59 % of the total who underwent surgical excision) was diagnosed with DCIS (Table 3). The lesion was initially seen as architectural distortion of 17 mm in size on the screening mammogram. The lesion was biopsied under breast MRI guidance using Vacuum-assisted 9 gauge needle with total of 5 core samples removed. The 25 patients who did not undergo surgical excision had stable imaging studies with mean (±SD) 26 (±20) months follow up.

**Table 3:** Cancer upgrade case after surgical excision

Image type	Lesion (size)	Biopsy method	Caliber of needle	# samples	Cancer type
Mammogram	Architectural distortion (17 mm)	MRI guided Vacuum Assisted	9 gauge	5 cores	DCIS

## Discussion

In the modern era of medicine, replacing surgical biopsy with image-guided core needle biopsy for non-palpable lesions initially discovered on screening is no longer controversial. The American College of Surgeons Commission on Cancer strongly supports this change in clinical practice. However, controversy still exists regarding when to surgically excise the residual lesion once core needle biopsy reveals proliferative benign breast disease, especially isolated RS. Spiculated radiologic characteristics of RS resemble malignancy, and it can be challenging to differentiate between this and tubular histotype carcinoma (López-Medina et al. 2006). Current literature on cancer upgrade rate after surgical excision widely varies from 0 to 40 % due to heterogeneous study patient populations, including mixture of isolated RS and RS with atypia lesions, study patient populations combining RS diagnosis from both core and upfront surgical excision biopsy, small study sample sizes, and lack of comparison between patients who did and did not undergo surgery (Resetkova et al. 2011; Linda et al. 2010; Sohn et al. 2010; Doyle et al. 2007; Douglas-Jones et al. 2007; Berg et al. 2008; Becker et al. 2006; Cawson et al. 2003; Brenner et al. 2002). Our study contributes to the literature by (1) evaluating a patient population consisting of only isolated RS without atypia and (2) comparing patients who did and did not undergo surgery after core needle biopsy. Sutter Medical Centers of Sacramento Sierra region serves over a million residents in both urban and suburban areas. The Sacramento Metropolitan Area is the largest in the Central Valley and the fourth largest in the state, and these service regions combined represents a study population that is generalizable to residents of northern California.

The cancer upgrade rates after surgery in our patient population with isolated RS found on core needle biopsy was 1.59 % (1/63) for DCIS. The histologic feature of DCIS was clearly different from tubular histotype histologically. There was no invasive carcinoma cancer upgrade.

The relatively low malignancy upgrade rate in this study supports the clinical judgment in favor of using large bore vacuum assisted core needle biopsy size, sampling at least 6 cores in the setting of lesion size less than 2 cm, and performing post biopsy re-imaging to evaluate residual disease after the core needle biopsy has been performed. If there is notable residual disease present or discordance, then surgical excision should be performed. Post biopsy residual disease on post biopsy imaging would be concerning for sampling error risk as studies suggest that the proportion of radial scar volume involved by carcinoma ranges from 3.7 to 16.2 % and carcinoma involved the periphery of the lesion (López-Medina et al. 2006).

Our radiologists perform approximately 4–6 core samples per lesion. On the other hand, Brenner et al. (2002) and Becker et al. (2006) recommends at least 12 core specimens using vacuum assisted large core needles (11 or 14 gauge) to achieve reliable samples. This difference is due to difference in patient population. Our patient population consists of radiological lesions with size less than 2 cm. Between 4 and 6 core samples for lesions less than 2 cm in size may be reliable based on our study findings, and is clinically outweighed by higher risk of bleeding, larger hematoma, and pain associated with greater number of core sampling case by case basis. This question requires further study in larger samples.

Finally, it is important to consider the 63 patients who underwent surgery who did not have cancer. While knowledge that the residual area has been completely excised and therefore ruled out for cancer is highly beneficial, it is also very important to question in what ways we are over-treating our patient population. There are potential complications associated with surgery such as wound dehiscence, infection, hematoma and pain. Also there are potential complications from anesthesia. There are personal costs, such as anxiety with associated surgery. Additional research studies to quantify these impacts is needed as well as additional study in other populations, such as different socio-demographic patient populations, different approaches to handling RS lesions, and different RS lesions radiologic presentations.

Advancement in breast imaging, pathology and multidisciplinary approaches to care may effectively guide non-surgical management of RS diagnosed on core needle biopsy. Based on our study findings, isolated radial scar on core needle biopsy may not warrant routine surgical excision given relatively low cancer upgrade rates. These conditions are met when there is an isolated radiologic estimation of RS less than 2 cm in size, radiology/pathology concordance, no associated atypia or LCIS, and no palpable lesion (Table 4). We recommend surgical excisional biopsy when there is a core needle biopsy showing RS associated with atypia or LCIS, radiology/pathology non-concordance, limited sampling (large lesion, bleeding during biopsy, notable residual disease), palpable lesion, or associated mass on US or breast MRI (Table 4).

**Table 4:** Isolated RS management recommendations

Surgical excisional biopsy recommendation	Imaging follow-up recommendation
Associated atypia or LCIS	Isolated RS <2 cm size
Radiology/pathology non-concordance	Concordance of radiology/pathology
Limited sampling (large lesion, bleeding during biopsy, notable residual disease)	No atypia or LCIS
Palpable lesion	Nonpalpable lesion
Associated mass on US or breast MRI	

## References

[CR1] Becker L, Trop I, David J, Latour M, Ouimet-Oliva D, Gaboury L (2006). Management of radial scars found at percutaneous breast biopsy. Can Assoc Radiol J.

[CR2] Berg JC, Visscher DW, Vierkant RA, Pankratz VS, Maloney SD, Lewis JT (2008). Breast cancer risk in women with radial scars in benign breast biopsies. Breast Cancer Res Treat.

[CR3] Brenner RJ, Jackman RJ, Parker SH, Evans WP, Philpotts L, Deutch BM (2002). Percutaneous core needle biopsy of radial scar of the breast: When is excision necessary?. AJR.

[CR4] Cawson JN, Malara F, Kavanagh A, Hill P, Balasubramanium G, Henderson M (2003). Fourteen-gauge needle core biopsy of mammographically evident radial scars: Is excision necessary?. Cancer.

[CR5] Degnim AC, Visscher DW, Berman HK, Frost MH, Sellers TA, Vierkant RA (2007). Stratification of breast cancer risk in women with atypia: a Mayo COHORT STUDY. J Clin Oncol.

[CR6] Douglas-Jones AG, Denson JL, Cox AC, Harries IB, Stevens G (2007). Radial scar lesions of the breast diagnosed by needle core biopsy: analysis of cases containing occult malignancy. J Clin Pathol.

[CR7] Doyle EM, Banville N, Quinn CM, Flanagan F, O’Doherty A, Hill AD (2007). Radial scars/complex sclerosing lesions and malignancy in screening programme; in cadence and histological features revisited. Histopathol.

[CR8] Dupont WD, Page DL (1985). Risk factors for breast cancer in women with proliferative breast disease. N Engl J Med.

[CR9] Howlader N, Noone AM, Krapcho M, Neyman N, Aminou R, Waldron W et al (2011) SEER cancer statistics review, 1975–2008. http://seer.cancer.gov/csr/1975_2008/_/csr/1975_2010/

[CR10] Krishnamurthy S, Bevers T, Kuerer H, Yang WT (2012). Multidiciplinary considerations in the management of high-risk breast lesions. AJR.

[CR11] Linda A, Zuiani C, Furlan A, Londero V, Girometti R, Machin P, Bazzocchi M (2010). Radial scars without atypia diagnosed at imaging-guided needle biopsy: How often is associated malignancy found at subsequent surgical excision, and do mammography and sonography predict which lesions are malignant?. AJR Am J Roentgenol.

[CR12] López-Medina A, Cintora E, Múgica B, Operé E, Vela AC, Ibañez T (2006). Radial scars diagnosed at steriotactic core-needle biopsy: surgical biopsy findings. Eur Radiol.

[CR13] Morgan C, Shah MC, Hamilton R, Wang J, Spigel J, DeLeon W (2012). The radial scar of the breast diagnosed at core needle biopsy. Proc (Bayl Univ Med Cent).

[CR14] Resetkova E, Edelweiss M, Albarracin CT, Yang WT (2011). Management of radial sclerosing lesions of the breast diagnosed using percutaneous vacuum-assisted cored needle biopsy: recommendations for excision based on seven years’ of experience at a single institution. Breast Cancer Treat.

[CR15] Saslow D, Boetes C, Burke W, Harms S, Leach MO, Lehman CD (2007). American cancer society guidelines for breast screening with MRI as an adjunct to mammography. CA Cancer J Clin.

[CR16] Sohn VY, Causey MW, Steele SR, Keylock JB, Brown TA (2010). The treatment of radial scars in the modern era–surgical excision is not required. Am Surg.

